# The Association of Four Natural Molecules—EGCG, Folic Acid, Vitamin B12, and HA—To Counteract HPV Cervical Lesions: A Case Report

**DOI:** 10.3390/jpm13030567

**Published:** 2023-03-22

**Authors:** Giovanni Grandi, Laura Botticelli, Pietro Di Fraia, Carla Babalini, Meris Masini, Vittorio Unfer

**Affiliations:** 1 Department of Medical and Surgical Sciences for Mother, Child and Adult, Obstetrics and Gynecology Unit, University of Modena and Reggio Emilia, Azienda Ospedaliero Universitaria Policlinico, 41125 Modena, Italy; 2 Department of Pathology, Azienda Ospedaliero Universitaria Policlinico, University of Modena and Reggio Emilia, 41125 Modena, Italy; 3 Di Fraia Laboratori s.r.l., 00034 Colleferro, Italy; 4 UniCamillus-Saint Camillus International University of Health Sciences, 00131 Rome, Italy

**Keywords:** HPV, cervical lesions, EGCG, vitamin B12, folic acid, HA

## Abstract

Precancerous lesions of the uterine cervix, due to HPV infections, are still today a great medical challenge. This clinical case highlighted the effectiveness of epigallocatechin gallate (EGCG), vitamin B12, folic acid, and hyaluronic acid (HA) in counteracting HPV lesions in a 39-year-old patient with a long history of viral persistence, cervical lesions of various degree, and several unsuccessful surgical approaches. After eight weeks of treatment, both the histological and cytological analyses revealed only a chronic cervicitis without any malignant lesions or cellular dysplasia, thus reducing the urgency of an invasive surgery, a total hysterectomy.

## 1. Introduction

The Human Papilloma Virus (HPV) is the etiologic agent of cervical cancer, the second most frequent tumor in women between 15 and 45 years of age [1]. Even though HPV infections concern both men and women, the high susceptibility of the cervical zone as a transformation zone between different kinds of epithelia, makes this region a “weak point” and an ideal tissue for the viral lifecycle [2]. Over 100 types of HPV exist, and among them, about 40 may infect the genital tract, mainly affecting the cervical transformation zone.

Depending on the oncogenic potential, papilloma viruses may be divided into low-risk (LR) and high-risk (HR). The former may cause benign external genital lesions (warts and condylomas), while the latter are associated with almost all types of cervical and anogenital cancers [3,4]. 

In about 80% of cases, the infection naturally reverts within one year due to the clearance activity by the immune system. However, in the remaining 20% of patients, the infection can persist over one year [5], thus facilitating viral integration in the host genome and promoting tumor progression [6]. The persistence of HPV is a great medical challenge, as it represents the evolutionary success of the virus, and it is the triggering cause of tumoral onset. In particular, undetected and untreated persistent infections due to HR types, especially HPV types 16 (HPV16) and 18 (HPV18), can cause almost all cervical cancers [7]. 

Quite often, the infection of HPV correlates with affections of cervical tissue, determining the occurrence of cervical intraepithelial lesions of various degrees, depending on the severity and the risk of tumoral progression. In detail, premalignant changes represent histological abnormalities ranging from Atypical Squamous Cells of Undetermined Significance (ASCUS) and mild dysplasia (CIN1) to moderate dysplasia (CIN2) and to severe dysplasia or carcinoma in situ (CIN3). Depending on the histological diagnosis and the degree of the severity, the treatment of the lesions is different. In the case of ASCUS/CIN1, physicians recommend no treatments at the time of diagnosis due to the high rate of spontaneous regression, while in the case of CIN2 or CIN3 they recommend surgical procedures. These latter include ablative methods that destroy the affected cervical tissue (cryotherapy, laser ablation, electrofulguration, and cold coagulation), as well as excisional methods, such as loop electrosurgical excision procedures (LEEPs) [8]. Nevertheless, postsurgical follow up of women with CIN2/3, treated with ablative or excisional methods, may be critical, since the treatment failure rates are estimated at 5–15% among all treatment modalities [9], and the recurrence rate may still remain quite high. 

In light of this, current treatments only target the clinical signs of the infection, such as condylomas or cervical lesions, but no specific therapies are available to eradicate the virus and its persistence. As of today, vaccines and screening programs are the only available tools to prevent HPV infection [10], reducing but not eliminating the risk of the progression of cervical cancer. Several studies have highlighted the antiproliferative and antitumoral activities of some natural molecules in the case of HPV infections. Among these, epigallocatechin gallate (EGCG), extracted from green tea, is a natural molecule well known for its antioxidant, anti-inflammatory, and antitumoral activity [11]. An in vitro study on a model of persistent HPV infection, demonstrated that EGCG had antiproliferative and proapoptotic activity in a dose-dependent way [12]. EGCG stimulated the increase in the expression levels of the proapoptotic marker p53 and reduced the levels of the viral oncoproteins E6/E7, which are responsible for the viral persistence, in a keratinocyte cell line containing episomal forms of HPV [13]. Another in vitro study demonstrated that EGCG could prevent cancer progression by promoting apoptosis in both HeLa and SiHa cells (HPV16-positive) [14,15]. A recent study further revealed the effect of EGCG on the interferon (IFN) pathway, which is one of the escape mechanisms of HPV. Pretreatment with EGCG of transfected keratinocytes with type 2 HPV (HPV2) E7 upregulated the IFN signaling pathway, thus reinforcing the innate antiviral immunity against HPV2 [16].

Moreover, a randomized clinical study from Ahn and colleagues investigated the efficacy of green tea extracts on 88 HPV positive women with squamous intraepithelial lesions of various degrees. The results revealed a significant improvement in the degree of the lesions after an 8/12-week treatment with 200 mg/day of oral EGCG [17]. 

Vitamin B12 and folic acid are crucial micronutrients whose deficiency may correlate with different pathological conditions, including cancer [18]. Both these micronutrients are involved in the methylation of the HPV genome, thus blocking both viral proliferation and persistence and increasing the clearance of the virus. In addition, the deficiency of both these micronutrients may predispose to DNA fragmentation, thus facilitating genomic integration of the HPV DNA [19]. Clinical evidence revealed that high levels of vitamin B12 and folic acid may reduce the risk of high-grade lesions’ progression toward a tumoral phenotype [20]. Moreover, high plasma levels of folic acid seem to have a protective effect, as they reduce the risk of HPV infection by 73% [21]. 

Hyaluronic acid (HA) is one of the most intriguing and versatile natural molecules. Despite its simple structure, HA has several functions according to its molecular weight. In particular, the reparative action of very low molecular weight HA (<5 kDa) [22] plays a crucial protective role as a physical barrier in the case of HPV infections. By restoring the integrity of the damaged epithelium or mucosae, due to microtraumas or scratches during sexual intercourse, HA may block HPV entry into the body. To date, indeed, the application of vaginal HA is one of the most used approaches to restore cervical re-epithelization and to support the spontaneous viral clearance, especially in conditions of low-grade squamous intraepithelial lesions (LSILs). A recent study revealed that supplementing HA in combination with other compounds may increase the viral clearance and reduce the persistence of LSIL/CIN1 lesions [23]. 

Despite several advances in scientific research and screening programs, HPV infection remains a global burden. Therefore, developing new therapeutical strategies to manage this infection and its persistence represents a great medical challenge. The aim of this clinical case was to describe the effect of the treatment with EGCG, vitamin B12, folic acid, and HA on the cervical lesions of a 39-year-old patient with indication for hysterectomy due to the recurrence of HPV infection.

## 2. Case Report

A 39-year-old patient with a 9-year clinical status of HPV persistence received a medical indication for hysterectomy. As it is an irreversible procedure and considering the fertile age of the patient, she decided to ask for another medical opinion, becoming the subject of this case report study. 

Regarding the background of the patient, her personal history reported she was a former smoker since 2016, while the obstetric and gynecological history revealed she had one physiological pregnancy with a cesarean delivery. In addition, she had no immunological diseases and she didn’t assume any immunosuppressive treatments. 

In 2013, a biopsy of the uterine cervix revealed a severe cervical intraepithelial neoplasia (CIN3), and she underwent loop electrosurgical procedure (LEEP). The histological analysis revealed a high-grade intraepithelial lesion (CIN2/3) with free margins followed by a persistent condition of low-grade squamous intraepithelial lesion (LSIL). In 2017, the HPV DNA test diagnosed the positivity for the high-risk HPV16. The next year, the Papanicolaou test (Pap-test) revealed a high-grade squamous intraepithelial lesion (HSIL). Later, the endocervical curettage and the relative tissue microscopical analysis revealed a condition of mild cervical intraepithelial neoplasia (LSIL/CIN1). Despite the clinical guidelines for LSILs, the patient underwent a second LEEP. Postsurgical analysis revealed no squamous intraepithelial lesions. Five months later, the Pap-test evidenced another HSIL, which was confirmed after 3 months. Postsurgical analysis after a third LEEP revealed a LSIL (CIN1) lesion. About 4 months later, another Pap-test revealed the presence of abnormal squamous cells in the tissue (ASCH) with a persistent positivity for HPV16, which was present for more than two years. Three months later, the HPV DNA test was still positive for HPV16. 

In 2020, seven years later from the initial diagnosis of HSIL (CIN3) and despite all the medical procedures, the patient still presented HSILs. Seven months later, the HPV positivity was still present, and the patient underwent cervicoscopy and hysteroscopy. The analysis revealed the presence of proliferating endometrial polypus and squamous intraepithelial lesions, which were consistent with a condition of HSIL (CIN3) with the margins of the resection free from lesion. The HPV DNA test was still positive for HPV16 type. Following these results, the patient underwent laser conization to remove the high-grade lesion. 

Five months later, the HPV16 positivity was still present, and the Pap-test revealed another HSIL. One month later, the patient repeated the colposcopy with biopsy. The objective gynecological exam revealed a regular vagina, without any alterations. The colposcopy reported that performing the exam was very difficult due to the anatomical condition of the uterus; the squamocolumnar junction (SCJ) was visible only back, and the transformation zone (TZ) grading was 1 back to the SCJ. So, physicians performed a biopsy that revealed again cytological atypia of low and high grade. 

At that time, following the troubled clinical history of the patient and considering her previous histological results and her anatomical condition, which did not allow further conservative interventions, the physicians proposed a surgery of total hysterectomy as the only solution. 

Therefore, following the request of the patient, we evaluated the effects of four natural molecules to explore alternative solutions to this invasive surgical procedure or at least to delay the need for this surgery.

## 3. Materials and Methods

### 3.1. Oral Dietary Supplementation

The patient underwent a treatment with the food supplement named Pervistop^®^ (Farmares s.r.l., Roma, Italy) in a regimen of two tabs/day for eight weeks. Each tablet contains 200 mg of EGCG, 50 mg of HA, 1 mg of vitamin B12, and 400 mcg of folic acid. 

### 3.2. Cervical–Vaginal Cytology (Pap-Test)

The exam of cervical–vaginal cytology is primarily a cervical cancer screening test. This test consists of a procedure that gently removes cells from the surface of the cervix and the surrounding area, and then perform the microscopical analysis. 

### 3.3. Histological Analysis

The histological analysis was performed by using the hematoxylin and eosin (H&E) staining. The hematoxylin stains cell nuclei purplish blue, while the eosin stains the cytoplasm and extracellular matrix pink. The pattern of the coloration shows the general layout and distributions of cells providing a general overview of the tissue structure. 

## 4. Results

After the eight-week supplementation, the young patient underwent a Pap-test and cervical biopsy for cytologic and histological analysis, respectively. 

The cervical–vaginal analysis reported an adequate picked-up sample, with the presence of endocervical tissue component. The medical response indicated a strong inflammation, but it was strikingly negative for intraepithelial or malignant lesions. In addition, the histological analysis of the cervical biopsy reported chronic cervicitis as the unique diagnosis, confirming the absence of atypia in the laminae of squamous epithelium. 

**Figure 1:**
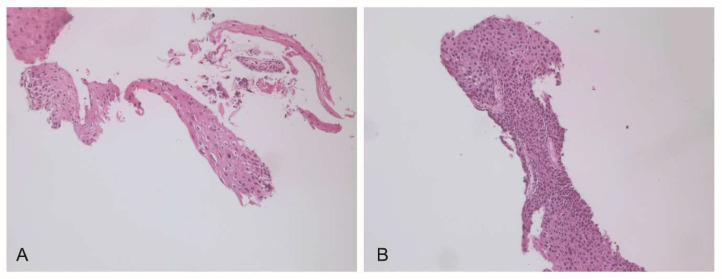
Histological analysis of the cervical tissue from biopsy before the treatment. The H&E staining of the cervical tissue indicates squamous intraepithelial lesions of varying degree. The presence of several koilocytosis and nuclear atypia (**A**) are a clear marker of LSIL; the higher proliferation of abnormal cells, nuclear atypia, and full thickness mitosis are evident in HSIL (**B**).

Before the treatment, the cervical biopsy revealed the presence of both LSIL and HSIL (Figure 1). 

Koilocytosis and nuclear atypia, associated with an ongoing viral infection, can be observed in the LSIL region (Figure 1A), while a higher proliferation of abnormal cells, nuclear atypia, and full thickness mitosis are associated with HSIL (Figure 1B). 

**Figure 2:**
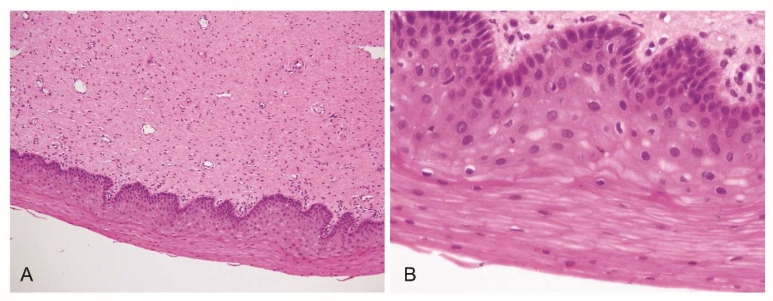
Histological analysis of the cervical tissue after the eight-week treatment. The H&E staining of the cervical tissue indicates, at different zoom levels (**A**, 10×; **B**, 40×), a chronic cervicitis with aspects of parakeratosis and focal cellular alterations of koilocytosis, without dysplasia.

Eight weeks after the oral assumption, the histological analysis highlighted parakeratosis and focal cellular alterations of koilocytosis attributable only to chronic cervicitis, without dysplasia (Figure 2A,B).

**Figure 3:**
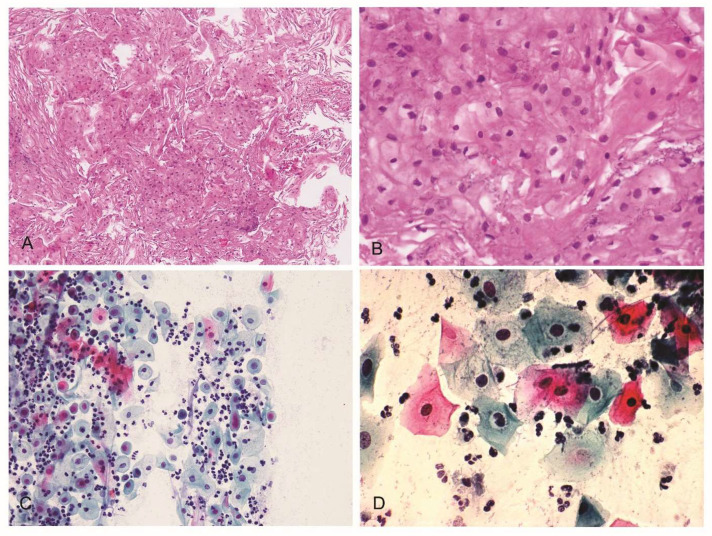
Histological and cytological analysis from the Pap-test after the eight-week treatment. The images of the scraping indicate, at different zoom levels, a squamous epithelial with focal perinuclear halos and hyperchromasia (**A**, 10×; **B**, 40×). The cytological analysis from the Pap-test, at different zoom levels, revealed some morphological alterations of the epithelial cells due to ongoing inflammatory processes (perinuclear halos, slightly hyperchromic or hypochromic nuclei, and a moderate increase in the volume of the nucleus) (**C**, 10×; **D**, 40×).

The analysis of the collected cervical scraping from the Pap-test showed a squamous epithelium with focal perinuclear halos and hyperchromatic nuclei (Figure 3A,B). The cytological analysis derived from the Pap-test revealed neither cellular or nuclear atypia nor abnormal proliferation. The cells appeared commonly arranged in small groups; several neutrophilic granulocytes and nuclear and cytoplasmatic detritus were evident in the collected sample (Figure 3C). Some morphological alterations of the epithelial cells indicated ongoing inflammatory processes, such as binucleation, perinuclear halos, slightly hyperchromic or hypochromic nuclei, and the moderate increase in the volume of the nucleus (Figure 3C,D).

Later, considering the complexity of the clinical history of the patient, she underwent a six-month follow up to monitor the HPV infection and the progression of cervical lesions. In particular, she underwent a colposcopy, Pap-test, and an HPV DNA test, and all the exams reported negative results. The objective gynecological exam reported regular external genitals and vagina, without alterations as regards the internal genital apparatus with linear endometrium and no effusion. The colposcopy did not observe any cervical lesions, and the Pap-test revealed no more cervical intraepithelial or malignant lesions. Finally, the HPV DNA test was also negative, thus confirming the improvement in the clinical conditions of the patient.

## 5. Discussion

In this clinical case, we demonstrated for the first time the effectiveness of a combination of four natural molecules—EGCG, vitamin B12, folic acid, and HA—in reducing the urgency of an invasive surgery, a total hysterectomy, in a young fertile patient. 

So far, clinical practice has had no tools for counteracting the persistence of the virus, since an effective treatment against the clearance of HPV is still lacking [24]. In the case of low-grade lesions, the general guidelines indicate only to monitor patients with recurring controls until they clear the infection or develop cervical neoplasia [25], while in the case of more serious and invasive lesions, surgical procedures are the best practice [26]. However, the available surgical treatments, which remove clinical manifestations of the infection, such as the cervical lesions, may not guarantee the clearance of the virus and the recurrence of new lesions, thus corroborating the existing gap in clinical practice [27,28]. Indeed, although some surgical techniques may be better than others in terms of reproductive and gynecological outcomes, none of them may strongly reduce the risk of recurrence. 

In the case of this 39-year-old patient, the HPV infection had persisted for nine years. She underwent surgical procedures, more or less invasive, but none of them was effective in eradicating the HPV infection, with the occurrence of new lesions. Therefore, physicians proposed the surgical remotion of the uterus as the only irreversible solution. 

The eight-week oral administration of EGCG, folic acid, vitamin B12, and HA improved her pathological condition; in fact, both the cytological and the histological analysis revealed no more squamous intraepithelial malignant lesions for the first time after nine years. The exams only reported a chronic cervicitis, without dysplasia or malignant events. The presence of some cellular morphological alterations indicated a strong inflammatory ongoing process, but no signs of malignant lesions appeared from the analysis, confirming the positive effects of the treatment. 

Both in vitro and in vivo pieces of evidence have already demonstrated the protective role of these natural molecules by blocking the progression of squamous intraepithelial lesions. Studies on the individual molecules demonstrated the efficacy of (i) EGCG as an antiproliferative and proapoptotic factor [12,13,14,15,16,17], (ii) vitamin B12 and (iii) folic acid as methylation agents that block viral proliferation and persistence [19], and (iv) HA as a physical barrier that restores the integrity of epithelium [22,23]. However, no studies, until this case report, have demonstrated their synergic action and especially their efficacy against the reappearance of cervical lesions. 

Here, we demonstrated that the association of four natural molecules had a protective effect in this complex HPV-pathological picture. They improved the existing cervical lesions, and they delayed the need for a hysterectomy, considered the only available solution for this patient. 

Subsequent exams, performed as a six-month follow up of the oral treatment, confirmed the initial beneficial results. The colposcopy, Pap-test, and HPV DNA test confirmed the effectiveness of the treatment, indicating that the oral assumption of these combined natural molecules may improve cervical intraepithelial lesions and contribute to a condition of HPV negativity. 

Although they are preliminary results reported at eight weeks and six months, this clinical case may pave the way for an innovative approach in the management of HPV cervical lesions and viral infection. Along with this, a recently published pilot clinical study also supports such positive effects. A small group of 20 women with persistent HPV infections and cervical lesions showed no cytological or histological evidence of lesions following the treatment with the same dosages of these four natural molecules [29].

In the meantime, the patient is still taking the therapy, without experiencing any adverse events, and longer monitoring will be necessary to assess the long-term beneficial effect of such treatment. 

## 6. Conclusions

The management of HPV infections remains a critical topic among physicians, especially considering the persistence of the virus with its evolutionary success. To date, current strategies only act on preventing HPV infections through vaccines and screening programs or on targeting the clinical manifestations, such as cervical lesions and condylomas. Specific treatments that counteract the viral persistence, eradicating the virus, are still lacking. 

In this clinical case, the eight-week oral supplementation, containing EGCG, vitamin B12, folic acid, and HA, restored the high-grade cervical lesions, thus preventing or delaying the surgery of hysterectomy in a fertile patient. 

In addition, the six-month follow up after the beginning of the oral assumption, corroborated the initial beneficial outcomes. The negativity of the HPV DNA test and the improved colposcopy examination, along with the objective gynecological exam, open the possibility of considering such results not casual but relative to the treatment.

Overall, in light of these results and considering (i) the antiproliferative and proapoptotic activities of EGCG, (ii) the preventive effect of vitamin B12 and (iii) folic acid against viral integration, and (iv) the re-epithelizing property of HA, the combination of these molecules provides a promising tool in the management of HPV infection. 

## Data Availability

Data will be available upon reasonable request.
